# Cell Surface Estrogen Receptor Alpha Is Upregulated during Subchronic Metabolic Stress and Inhibits Neuronal Cell Degeneration

**DOI:** 10.1371/journal.pone.0042339

**Published:** 2012-07-31

**Authors:** Cristiana Barbati, Marina Pierdominici, Lucrezia Gambardella, Fiorella Malchiodi Albedi, Richard H. Karas, Giuseppe Rosano, Walter Malorni, Elena Ortona

**Affiliations:** 1 San Raffaele Institute Sulmona, L'Aquila, Italy; 2 Department of Cell Biology and Neurosciences, Istituto Superiore di Sanità, Rome, Italy; 3 Department of Therapeutic Research and Medicine Evaluation, Istituto Superiore di Sanità, Rome, Italy; 4 Molecular Cardiology Research Institute, Tufts Medical Center, Boston, Massachusetts, United States of America; 5 Department of Medical Sciences, IRCCS San Raffaele Pisana, Rome, Italy; Max Planck Institute of Psychiatry, Germany

## Abstract

In addition to the classical nuclear estrogen receptor, the expression of non-nuclear estrogen receptors localized to the cell surface membrane (mER) has recently been demonstrated. Estrogen and its receptors have been implicated in the development or progression of numerous neurodegenerative disorders. Furthermore, the pathogenesis of these diseases has been associated with disturbances of two key cellular programs: apoptosis and autophagy. An excess of apoptosis or a defect in autophagy has been implicated in neurodegeneration. The aim of this study was to clarify the role of ER in determining neuronal cell fate and the possible implication of these receptors in regulating either apoptosis or autophagy. The human neuronal cell line SH-SY5Y and mouse neuronal cells in primary culture were thus exposed to chronic minimal peroxide treatment (CMP), a form of subcytotoxic minimal chronic stress previously that mimics multiple aspects of long-term cell stress and represents a limited molecular proxy for neurodegenerative processes. We actually found that either E2 or E2-bovine serum albumin construct (E2BSA, i.e. a non-permeant form of E2) was capable of modulating intracellular cell signals and regulating cell survival and death. In particular, under CMP, the up-regulation of mERα, but not mERβ, was associated with functional signals (ERK phosphorylation and p38 dephosphorylation) compatible with autophagic cytoprotection triggering and leading to cell survival. The mERα trafficking appeared to be independent of the microfilament system cytoskeletal network but was seemingly associated with microtubular apparatus network, i.e., to MAP2 molecular chaperone. Importantly, antioxidant treatments, administration of siRNA to ERα, or the presence of antagonist of ERα hindered these events. These results support that the surface expression of mERα plays a pivotal role in determining cell fate, and that ligand-induced activation of mER signalling exerts a powerful cell-survival signal. These results shed new light on the pathogenetic mechanisms leading to neuronal cell degeneration.

## Introduction

Several lines of evidence indicate that 17β-estradiol (E2) directly modulates the development and function of neurons, although the mechanism(s) by which this might occur is not well understood [1]–[3]. The primary mechanism of E2 activity is mediated by transcriptional actions of the intracellular, nuclear estrogen receptors (nER), ERα and ERβ, to produce genomic effects. A variety of cellular responses to physiological concentrations of E2 occurs rapidly, within seconds to few minutes, so that they cannot be mediated by transcription and protein synthesis. These rapid estrogen-mediated effects (referred to as “nongenomic”) are triggered through the activation of non-nuclear membrane-associated ER (mER) [4]–[8]. These receptors are structurally similar to their intracellular counterparts and, after ligand binding, they activate various protein kinase cascades, including extracellular signal-regulated kinase/mitogen-activated protein kinase (ERK/MAPK), protein kinase A, protein kinase C, Akt, and phosphatidylinositol 3-OH kinase (PI3K) [5]. The effects of E2 in the brain are mainly mediated by the nuclear ER-mediated genomic signaling pathway, which seems to exert a cytoprotective activity, e.g. increasing the expression of the anti apoptotic molecule Bcl-2 in hippocampal neurons in culture [9]–[11]. In addition, it has been suggested that the E2-dependent nongenomic signaling, by hindering apoptotic cell death, mediates neuroprotection and preservation of cognitive function following global cerebral ischemia, supporting a potentially important role of non-nuclear mER [12]. In this regard, the expression levels of mERα and mERβ, acting independently from nuclear ER, have been demonstrated to trigger a functional and prompt cell response. This appears to play a key role in mediating estrogen's effects: an increased expression of mERα has to be considered as protective whereas an increased expression of mERβ leads to cell demise [13]. In particular, a decreased ERα∶ERβ ratio might trigger a rapid phosphorylation of p38 MAPK, which in turn phosphorylates the p53 tumor suppressor and accelerates apoptosis rate [14]. A further actor in this complex scenario is represented by autophagy, a cytoprotective mechanism characterized by the ability of the cell to respond to metabolic stress by recycling damaged materials or organelles in vacuoles, i.e. autophagolysosomes, and leading to cell survival [15]–[19]. Autophagic vacuolar flux is precisely regulated by a complex cascade of events and also involves ERK/MAPK and p38 pathways, two well-known estrogen-activated signaling cascades [20].

Apart from physiological processes, estrogen has also been implicated in the development or progression of numerous neurodegenerative disorders, including stroke, Alzheimer's disease and Parkinson's disease. Although the precise role of ER in these diseases has scarcely been investigated, either apoptosis or autophagy have been suggested to play a pathogenetic role [21]. The aim of this study is to clarify the role of intracellular and cell surface ER in determining neuronal cell fate and the possible implication of these receptors in regulating either apoptosis or autophagy in neuronal cells under chronic minimal peroxide (CMP) treatment. This is a form of subcytotoxic minimal chronic stress recently proposed as a useful tool for the investigation of neuronal cell degeneration pathways [22]. In fact, CMP treatment at multiple levels is able to e strongly recapitulate multiple aspects of long-term cell stress and as such, it represents a limited molecular proxy for neurodegenerative processes [22]. We first investigated the expression of intracellular (nER) and cell surface ERα and ERβ in two cell models, i.e., the human neuronal cell line SH-SY5Y and primary cultures of rat hippocampal neurons, both representing key cell systems for the studies on neurodegenerative diseases. We then investigated whether under protracted, tolerable oxidative injury, such as CMP, E2 and E2-albumin construct (E2BSA), the non-permeant form of E2, were capable of modulating cell signals and regulating cell survival or death.

## Results

### CMP induces up-regulation of surface ERα

**Figure 1:**
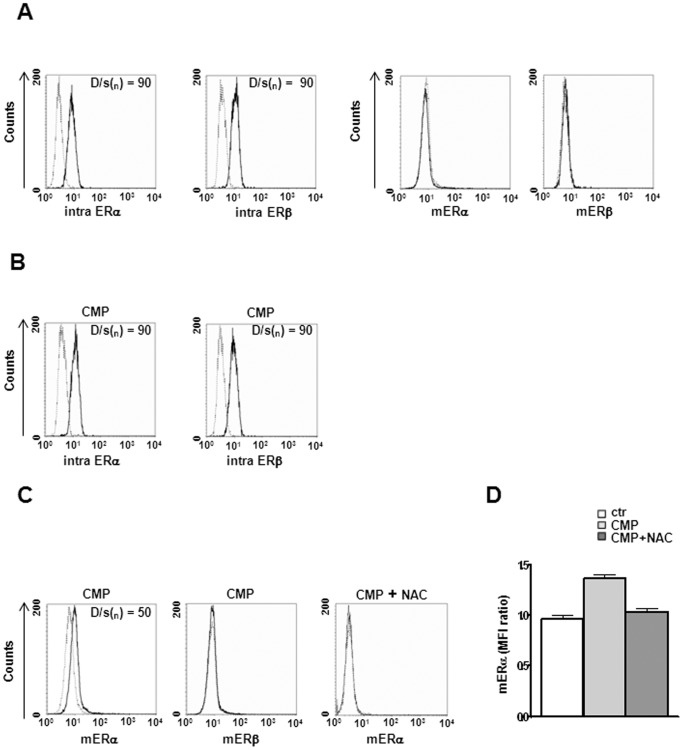
Flow cytometry analysis of intracellular and cell surface ER expression in SH-SY5Y neuronal cells. Representative flow cytometry histogram plots show the fluorescence intensity of MC-20 and 1531 Abs for ERα and ERβ, respectively, compared with isotype controls, in (**A**) untreated cells (intracellular ER, left panels and mER, right panels), (**B**) CMP treated cells (intracellular ER), (**C**) CMP or CMP+NAC treated cells (mER). Dotted lines indicate isotype control staining and solid lines indicate anti-ERα- and anti-ERβ-labeled cells. Statistical differences between the peaks of cells were evaluated by the Kolmogorov-Smirnov test (a D/s_(n)_ ratio ≥15 was accepted as significant). (**D**) Values of mERα/isotype control mean fluorescence intensity (MFI) *ratio* are reported. The mean ± SD from 5 independent experiments is shown. Ctr, untreated cells.

**Figure 2:**
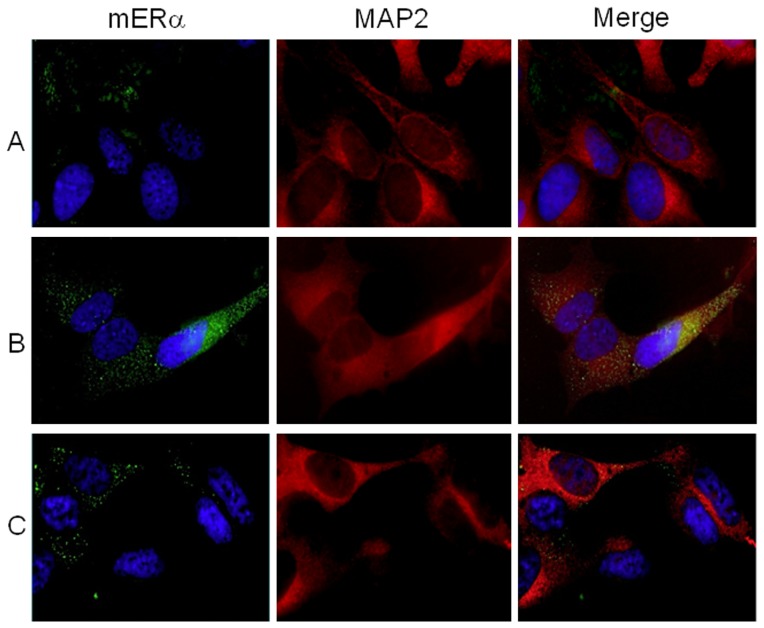
Immunofluorescence analysis of cell surface mERα in SH-SY5Y cells. mERα expression (green fluorescence) in unpermeated SH-SY5Y cells and MAP2 expression (red fluorescence) in Triton X100 permeated cells: (**A**) untreated, (**B**) CMP or (**C**) NAC/CMP treated cells. Cells were counterstained with Hoechst dye to reveal nuclei (blue staining). Note the yellow spots suggesting a colocalization of ERα and MAP2 in CMP exposed cells. No yellow spots are instead observable in untreated and NAC/CMP treated cells. Magnification: 3500×.

**Figure 3:**
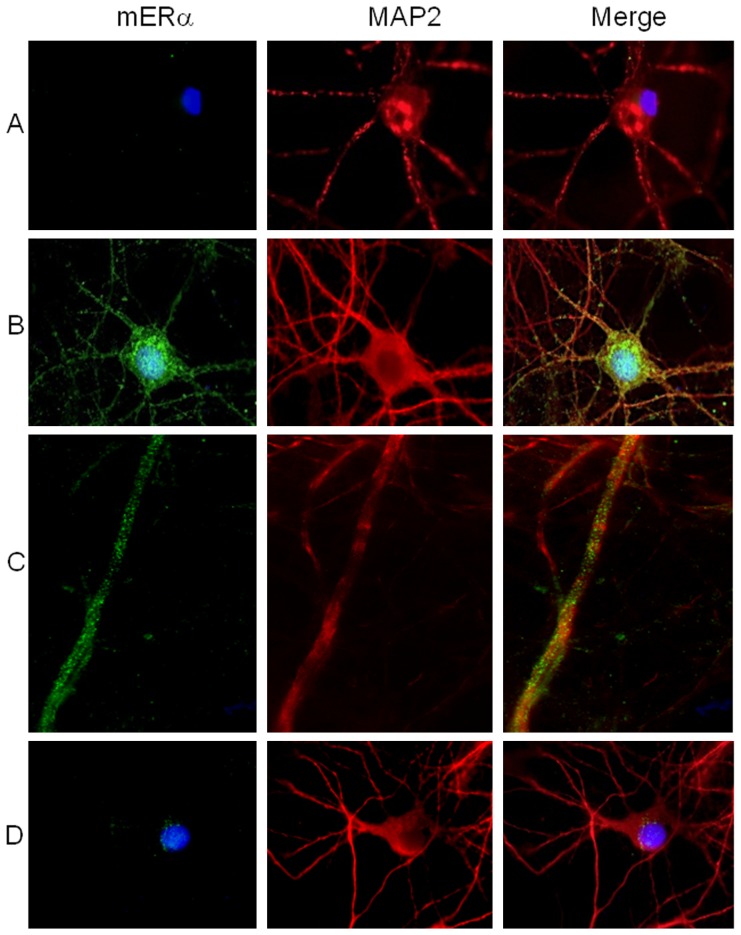
Immunofluorescence analysis of cell surface mERα in rat hippocampal neurons. mERα expression (green fluorescence) in unpermeated hippocampal neuronal cells and MAP2 expression (red fluorescence) in Triton X100 permeated hippocampal neuronal cells: (**A**) untreated, (**B, C**) CMP treated or (**D**) NAC exposed before CMP treatment. Note the yellow spots in (**B**) and (**C**) suggesting a colocalization of ERα and MAP2 in soma (**B**) and dendritic protrusions (**C**) of CMP exposed cells. No yellow spots are instead observable in untreated and NAC/CMP exposed hippocampal cells. Cells were counterstained with Hoechst dye to reveal nuclei (blue staining). Magnifications: (**A**) (**B**) (**D**) 3500×; (**C**) 6000×.

We first investigated the expression of ER in physiological condition and after a chronic minimal peroxide (CMP) stress as stated elsewhere [22] (see Materials and Methods). Flow cytometry analysis clearly showed that both the isoforms of ER, i.e., ERα and ERβ, were expressed at intracellular level in SH-SY5Y neuronal cells without significant differences between each other (positivity for ER expression was evaluated using the Kolmogorov-Smirnov analysis, **Figure 1A**, left panels). CMP treatment did not influence intracellular ER expression (**Figure 1B**). On the basis of literature demonstrating the presence of functional ER at the surface membrane of cells of different histotypes [23]–[26], we evaluated the cell surface expression of mER. This analysis revealed that both mERα and mERβ were undetectable on SH-SY5Y untreated cells (**Figure 1A**, right panels). Conversely, under CMP-induced stress, mERα expression was observed on SH-SY5Y cells whereas mERβ expression remained undetectable (**Figure 1C,D**). Since CMP induced reactive oxygen species (ROS) generation and a mild oxidative imbalance in the cell cytoplasm [22], we decided to evaluate the possible implication of ROS generation in the up-modulation of mERα by pre-treating SH-SY5Y cells with N-acetyl-L-cysteine (NAC). This compound is known to exert an antioxidant activity by acting as a thiol group supplier [27]. The results of these experiments clearly indicated that pre-treatment of cells with NAC before CMP-induced stress prevented CMP-induced upregulation of mERα (**Figure 1C,D**). The mERα expression was also evaluated by immunofluorescence microscopy at high magnification in unpermeated cells (**Figure 2**). Consistent with the results obtained by flow cytometry, immunofluorescence microscopy failed to reveal mERα in untreated cells (**Figure 2A**), whereas clearly displayed the presence of mERα at the surface of CMP-treated cells (**Figure 2B**, note the punctate green fluorescence). Additionally, mERα expression was markedly reduced in NAC/CMP-treated cells (**Figure 2C**). Since SH-SY5Y cells have to be considered as highly proliferating cells of tumor origin, we further evaluated the expression of mERα in differentiated neurons (primary cultures of rat hippocampal neurons at Day-In-Vitro, DIV, 14). Hippocampal neurons at DIV 14 are highly polarized cells, with distinct compartmentalization of cytoskeletal elements in the dendritic and axonal domains. Similar results were obtained with these primary cells compared to those obtained with SH-SY5Y cells, in that the surface expression of mERα was undetectable in untreated (**Figure 3A**) and in NAC/CMP-treated (**Figure 3D**) cells and detectable in CMP-treated cells (**Figure 3B,C**). Since recent evidence highlights the relationship between extra-nuclear signaling of ERα and the cytoskeleton in the central nervous system [28], we investigated the expression of cytoskeleton proteins and their possible colocalization with mERα in neuronal cells under CMP treatment. In particular, we labeled these cells for neuronal-specific microtubule associated protein (MAP)2 [29] and actin microfilaments. Both SH-SY5Y cells and rat hippocampal neurons were positive to MAP2 (**Figure 2A** **and** **3A** respectively, red fluorescence) and the expression of this protein was partially overlaid to that of mERα (yellow spots, **Figure 2B** **,** **3B**). Of note, the mERα expression was particularly evident in MAP2-positive somatodendritic compartment (**Figure 3C**). Control experiments were also performed on actin microfilaments, which allowed us to assess the integrity of neuritic tree. CMP treatment did not induce alteration of the dendritic trees although some actin filament interruptions and fragmentations were detectable (**Figure S1**). No positivity for mERα was detectable along non-dendritic actin filaments.

### mERα modulates cell survival signals

**Figure 4:**
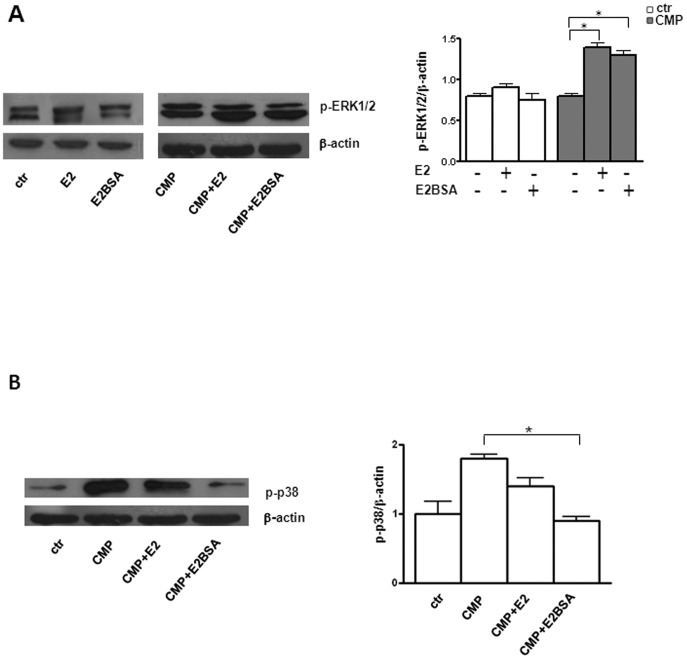
Effect of E2BSA and E2 on the activation of ERK and p38 MAPK. (**A**) Western blot analysis of p-ERK1/2 in untreated and CMP treated SH-SY5Y cells in presence or absence of estrogens. To ensure the presence of equal amounts of protein, the membranes were re-probed with a mouse anti-β-actin monoclonal Ab. Data from one representative experiment out of 10 are shown (left panel). Densitometric analysis of p-ERK1/2 levels relative to β-actin is also shown (right panel). Values are expressed as means ± SD from 10 independent experiments. *, *P*<0.01. (**B**) Western blot analysis of p-p38 in untreated and CMP treated neuronal cells in presence of absence of estrogens. Data from one representative experiment out of 10 are shown (left panel). Densitometric analysis of p-p38 levels relative to β-actin is also shown (right panel). Values are expressed as means ± SD from 10 independent experiments. *, *P*<0.01. Ctr, untreated cells.

Based on the results reported above, an important point was to evaluate whether mER were functional receptors. To provide evidence of a signaling function for estrogen *via* mER on neuronal cells, we determined in SH-SY5Y cells the effect of a membrane non-permeant form of E2 (E2BSA) and, as a control, of a membrane permeant E2 form, on the activation of two key molecules involved in protein kinase cascades regulating cell homeostasis: ERK and p38 MAPK. The first is involved in functions including the regulation of mitosis and post-mitotic functions in differentiated cells [30], whilst the second is involved in cell differentiation and apoptosis [31]. We found that in physiological conditions, estrogen (either E2BSA or E2) did not affect ERK phosphorylation (p-ERK) level (**Figure 4A**). Conversely, under CMP treatment both non-permeant and permeant forms of E2 significantly increased p-ERK levels (ratio p-ERK/β-actin, 0.8±0.05% *vs* 1.3±0.1 for E2BSA treatment, and 0.8±0.05 *vs* 1.4±0.1 for E2 treatment P<0.01 in both cases, see **Figure 4A**). Regarding p38 phosphorylation (p-p38), treatment with both non-permeant and permeant forms of E2 did not influence p-p38 level (data not shown). Interestingly, E2BSA exposure (30 min) was capable of preventing the increase of p-p38 induced by CMP treatment (ratio p-p38/β-actin, 1.8±0.1% *vs* 0.8±0.1, P<0.01, **Figure 4B**).

### Estrogen effects on cell fate

Based on the results reported above, we decided to evaluate whether estrogen, acting through the mERα, could modulate two major cell processes determining cell survival or death: apoptosis and autophagy.

### mERα ligation hinders apoptosis

**Figure 5:**
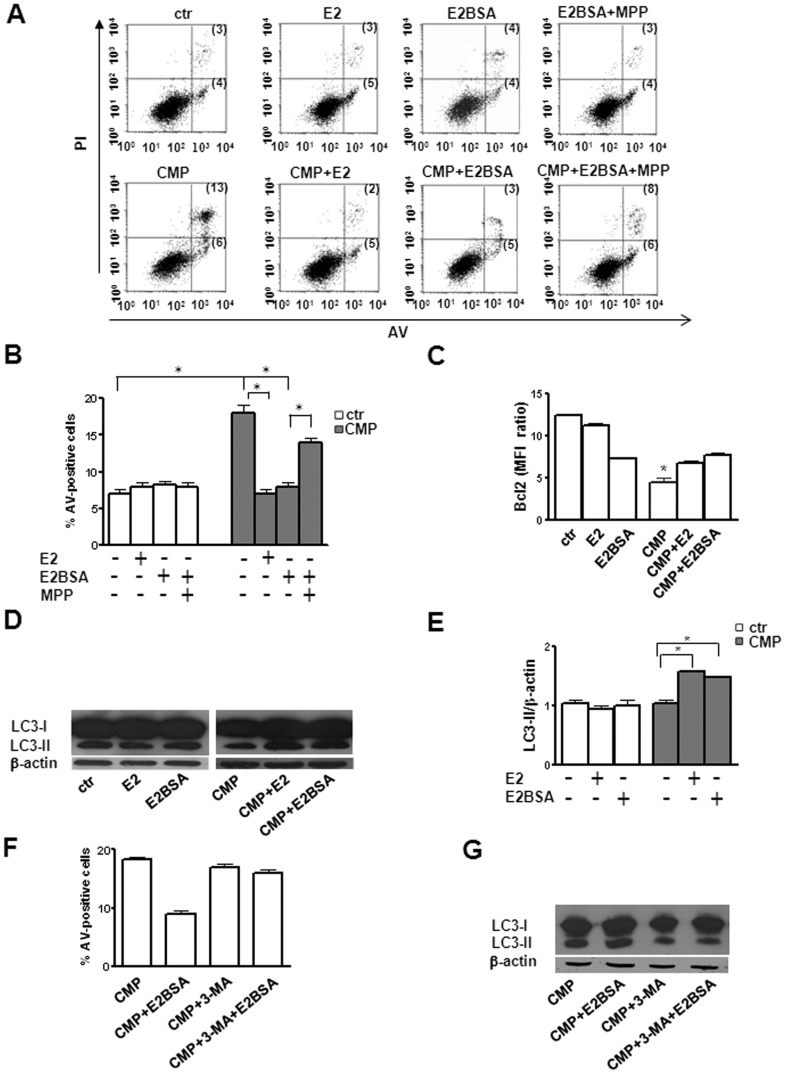
Effect of E2BSA and E2 on the modulation of apoptosis and autophagy. (**A**) Flow cytometry analysis of apoptosis in untreated and CMP treated SH-SY5Y cells in presence or absence of E2BSA, E2 and ERα antagonist MPP. Results obtained in a representative experiment are shown. Numbers in upper and bottom right quadrants of each plot refer to AV/PI double positive cells and to AV single positive cells, respectively. (**B**) Mean ± SD of the percentages of AV positive cells obtained in 10 independent experiments is reported. * *P*<0.001. (**C**) Values of MFI *ratio* (Bcl-2/isotype control) are reported. The mean ± SD from 10 independent experiments is shown. * *P*<0.01 for CMP treated cells *vs* all other conditions. Ctr, untreated cells. (**D**) Western blot analysis of LC3-II in untreated and CMP treated SH-SY5Y cells in presence or absence of E2 or E2BSA. Blots shown are representative of 10 independent experiments. (**E**) Densitometry analysis of LC3-II levels relative to β-actin is shown. Values are expressed as mean ± SD. * *P*<0.01. Ctr, untreated cells. (**F**) Flow cytometry analysis of apoptosis in untreated and CMP treated SH-SY5Y cells in presence or absence of the autophagy inhibitor 3-MA and E2BSA. Mean ± SD of the percentages of AV positive cells obtained in 3 independent experiments is reported. (**G**) Western blot analysis of LC3-II in CMP treated SH-SY5Y cells in presence or absence of the autophagy inhibitor 3-MA and E2BSA. Blots shown are representative of 3 independent experiments.

We evaluated the role of estrogen in modulating apoptosis in different experimental conditions (**Figure 5**). We found that in physiological conditions E2 and E2BSA did not affect apoptosis of SH-SY5Y cells. By contrast, after CMP treatment, the percentage of apoptosis, as expected, was significantly increased (P<0.001, **Figure 5A,B**), although the subcytotoxic experimental conditions used here did not affect cell proliferation and cell cycle progression (**Figure S2**). Interestingly, estrogen exposure was capable of preventing the increase of apoptosis induced by CMP treatment (18±2% *vs* 8±1% for E2BSA treatment and 18±2% *vs 7*±1% for E2 treatment, P<0.01 in both cases, **Figure 5A,B**). We also analyzed the expression of the key anti-apoptotic protein Bcl2. Concordant with the above results, we found that the level of this protein (expressed as mean fluorescence intensity, MFI) was significantly decreased in SH-SY5Y cells under CMP treatment in comparison with untreated cells (P<0.01), but both E2BSA and E2 were able to prevent this decrease (P<0.01, **Figure 5C**). Hence, mERα activation can support functional survival signals inside the cells.

### mERα ligation induces autophagy

Autophagy is an essential cytoprotective mechanism contributing to neuronal cell homeostasis [32]. A critical event in autophagy is the conversion of microtubule-associated protein 1 light chain 3 (LC3) from the free LC3-I form to the membrane-bound LC3-II form. Therefore the level of this form of LC3 is a good quantification for autophagy [33]. In physiological conditions, we found that estrogen did not affect the LC3-II levels in SH-SY5Y cells (**Figure 5D,E**). After CMP treatment LC3-II appeared unchanged, but it significantly increased after both non-permeant and permeant estrogen exposure (ratio LC3-II/β-actin, 1±0.2 *vs* 1.5±0.3 under CMP and E2BSA treatment and 1±0.2 *vs* 1.6±0.3 under CMP and E2 treatment, P<0.01 in both cases, **Figure 5D,E**). To demonstrate that estrogen administration can induce autophagy as cytoprotection mechanism, we inhibited autophagy using 3-methyl adenine (3-MA), an inhibitor of type III phosphatidylinositol 3-kinases that blocks autophagosome formation [34]. In the presence of 3-MA, E2BSA failed to protect cells under CMP treatment from cell death so that “normal” apoptotic susceptibility was restored (**Figure 5F**). As expected, in CMP-treated SH-SY5Y cells, E2BSA in presence of 3-MA did not induce the increase of LC3-II level (**Figure 5G**).

### mERα inhibition and knockdown hinders estrogen mediated effects

**Figure 6:**
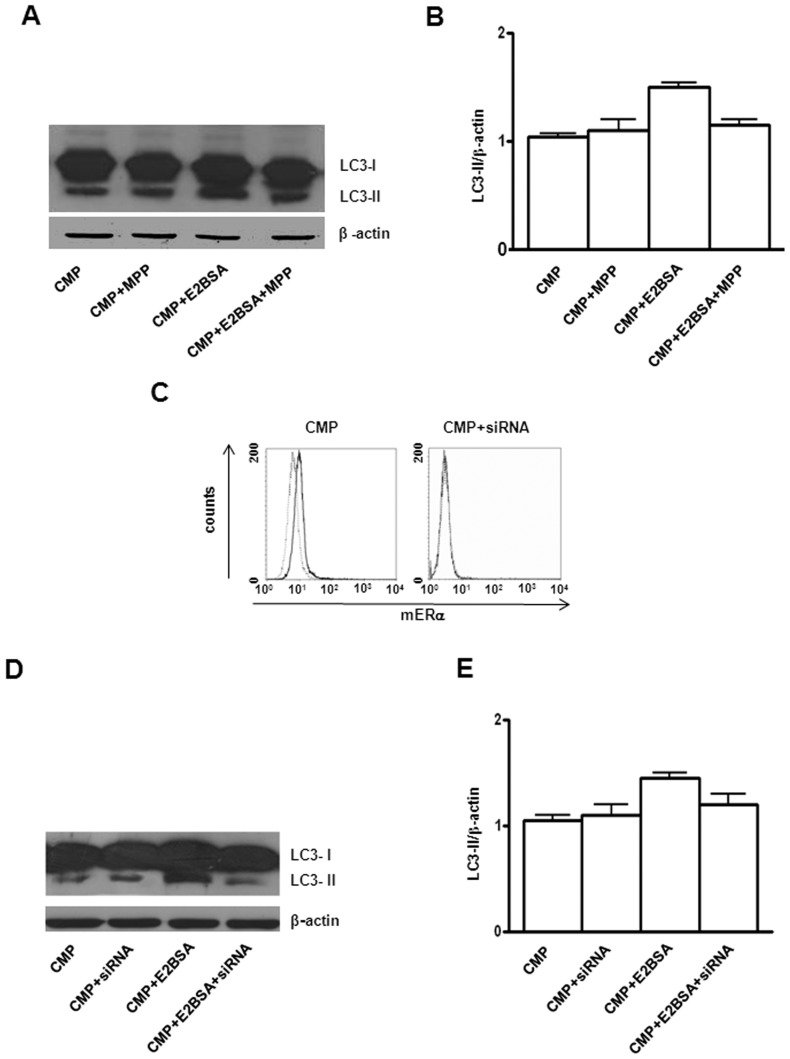
Effects of ERα inhibition and knockdown. (**A**) Western blot analysis of LC3-II in CMP treated SH-SY5Y cells in presence or absence of E2BSA and ERα inhibitor MPP. Blot shown is representative of 3 independent experiments. (**B**) Densitometry analysis of LC3-II levels relative to β-actin is shown. Values are expressed as mean ± SD. (**C**) Flow cytometry analysis of cell surface mERα expression 48 h after siRNA transfection. Isotype control staining is represented by the dotted line and anti-ERα-labeled cells are represented by the solid line. A representative experiment out of three is shown. (**D**) Western blot analysis of LC3-II in CMP treated SH-SY5Y cells and in silencing CMP treated SH-SY5Y cells in presence or absence of E2BSA. Blot shown is representative of 3 independent experiments. (**E**) Densitometry analysis of LC3-II levels relative to β-actin is shown. Values are expressed as means ± SD.

Finally, in order to clarify the role of mERα in estrogen-mediated autophagy, we treated SH-SY5Y cells with a specific ERα antagonist (methyl-piperidino-pyrazole, MPP) or transfected them with ERα siRNA. Pre-treatment with MPP inhibited the protective activity exerted by E2BSA, restoring apoptotic susceptibility (**Figure 5B**). Fittingly, MPP treatment also decreased significantly E2BSA-induced autophagy, detectable under CMP treatment (**Figure 6A,B**). In ERα silencing experiments, we found a significant reduction of mERα surface expression in transfected cells with respect to non-silencing siRNA transfected cells (MFI for mERα expression: 1.4±0.2 *vs* 1.1±0.2, P<0.01, **Figure 6C**). In CMP-treated cells, knocking down ERα significantly attenuated E2BSA-mediated enhancement of autophagy (1.4±0.1 vs 1.2±0.2 P<0.01, **Figure 6D,E**). These results suggest a regulatory role for mERα in cell “fate” modulation.

## Discussion

In this study, we show that under a condition of protracted, tolerable oxidative imbalance, ERα, but not ERβ, was expressed at the surface of SH-SY5Y, a neuroblastoma cell line considered as a paradigmatic model of dopaminergic neurons, and of rat hippocampal primary neurons. Importantly, this stress-associated upregulation of mERα at the cell surface could provide important survival signals to the neuronal cells. Its agonist, i.e. non-permeant E2, was in fact able to trigger various intracellular signals, including ERK phosphorylation and p38 inhibition, leading to an inhibition of apoptotic cell death and igniting cell cytoprotection by autophagy. The abrogation of these effects by pretreatment with an ERα antagonist and by transfection with specific siRNA clearly indicated the involvement of ERα in the neuroprotective mechanism mediated by estrogens.

The first point to consider is that, under CMP, neuronal cells can up-regulate mER. In particular, under chronic sublethal stress somewhat mimicking chronic inflammation, neuronal cells express at their surface mERα but not mERβ. This could be relevant in the maintenance of cell homeostasis. In fact, while mERα ligation has been hypothesized to be associated with cell survival signals, mERβ ligation was associated with cell death by apoptosis [13]. This would be consistent with a variety of other circumstances where ERα and ERβ have been shown to exhibit opposing actions. Hence, it seems conceivable that the mERα up-regulation detectable in CMP-treated cells could provide the cell with a mechanism to counteract subcellular modifications triggered by CMP conditions, including mild oxidative alterations. In fact, administration of NAC, which is known to be capable of exerting an antioxidant activity by replenishing the intracellular thiol pool, e.g. reduced glutathione (GSH), clearly inhibited mERα up-regulation.

Sex steroids are effective regulators of cell morphology and tissue organization, and recent evidence indicates that this is obtained through the regulatory activity of the cytoskeletal network [28]. Intriguingly, many of these regulatory actions related to cell morphology are achieved through rapid, non-classical signaling of sex steroid receptors to kinase cascades, independently from a direct nuclear alteration of gene expression or protein synthesis. For instance, we found that the expression of mERα at the cell surface, occurring after CMP, was associated with the expression of the MAP2 cytoskeletal protein, known to be involved in microtubule assembly and neurogenesis [29]. We cannot rule out the possibility that MAP2 phosphorylation and overexpression, previously observed in E2-treated hippocampal cells [35]–[37], could play a role in CMP-associated mERα expression at the cell surface. Conversely, actin microfilaments, although partially rearranged in CMP-treated neuronal cells, did not display any overlay with mERα expression, so that a role for actin filaments in mERα up-regulation should be ruled out.

We demonstrated the functional activity of the cell surface mERα by treatment of neuronal cells with E2BSA under CMP-mediated stress. The ligation of mERα activates intracellular signals, increasing pERK phosphorylation and decreasing p-p38 levels. These results are in line with recent findings that support the hypothesis that mER represent functional receptors in different cell types, including blood vessel cells, lymphocytes or neuronal cells [5]. For instance, in neuronal cells, mER can affect the regulation of key proteins involved in neurotransmitter release at synapses, i.e. synapsins, via cytoskeleton [38]. We actually found that under unfavorable conditions, i.e. mimicking an inflammatory microenvironment, the up-modulation of the expression of mERα at the cell surface could represent an important controller of cell fate. In fact, we found that mERα ligation by E2BSA was capable of influence cell cytoprotection by lowering apoptosis rates and bolstering autophagic pathway. Accordingly, recent lines of evidence suggested the possible role of autophagy as cytoprotection mechanism counteracting cell demise by apoptosis in neurodegenerative disorders [39].

In conclusion, in line with some recent literature data [40], [41], our results highlight the importance of mERα (and estrogens) in regulating the coordinated framework of defense strategies aimed at counteracting chronic oxidative imbalance, a causative factor of neuronal cell aging and degeneration, and in determining cell fate.

## Materials and Methods

### Cell culture and treatments

SH-SY5Y neuroblastoma cells were grown in Dulbecco's modified Eagle's medium (DMEM) supplemented with 10% heat-inactivated fetal calf serum (FCS) (Euroclone, Pero, Milan, Italy), and 50 µg/mL gentamicin (Sigma, St Louis, MO) in a humidified air 5% CO_2_ chamber at 37°C. Cells of passage number 5–15, from ATCC, were used for all experiments to prevent any alteration of growth or response phenotype.

Primary hippocampal neurons were prepared from embryonic-day-18 rat brain, according to the method of Brewer et al. [42], slightly modified [43]. All experimental procedures were in line with the “Ethical principles and guidelines for scientific experiments on animals” of the Swiss Academy of Medical Sciences and national laws. After dissection, the hippocampi were treated with 2.5% trypsin and dissociated. The cells were plated in 24-well plates, containing poly-l-lysine-treated glass coverslips, in MEM, containing 10% heat-inactivated FCS (Euroclone). After 2 h, the cell culture medium was substituted with Neurobasal medium with B27 supplement (NBM/B27, Invitrogen, Carlsbad, CA), 700 µl for well. At Day-In-Vitro (DIV) 1, 5 µM arabinosylcytosine was added. Neuronal cell cultures contained less than 1% of astrocytes, as shown by glial fibrillary acidic protein staining (data not shown). Hippocampal neurons were used at DIV14 (mature neurons).

To induce protracted, tolerable oxidative stress, named chronic minimal peroxide (CMP) treatment, cells were treated with 10 nM H_2_O_2_ (Sigma) for five-seven days. Medium and hydrogen peroxide were changed daily. Cells were incubated with E2 (Sigma) and with membrane-non-permeant E2BSA (molar ratio E2∶BSA = 30∶1; Sigma) at a concentration of 10 nM (physiological concentration) for 48 h and 30 min, respectively. In separate experiments, cells were pre-treated with i) 10 mM NAC for 2 h; ii) 100 nM MPP ERα antagonist (Tocris Cookson, Ellisville, MO) for 1 h. For inhibition of autophagy, cells were treated with 5 mM 3-MA (Sigma) for 16 h before lysis of the cells for LC3II detection (see below).

### Flow cytometry analysis

For evaluating cell surface expression of ER, cells were stained for 30 min at 4°C with the following antibodies (Abs): rabbit anti-human ERα MC-20 policlonal Ab, directed to the C-terminus of the ERα; mouse anti-human ERβ 1531 monoclonal Ab, directed to the C-terminus of the ERβ. These antibodies were purchased from Santa Cruz Biotechnology (Santa Cruz, CA) and 1 µg per sample (1×10^6^ cells) was used. Equal amounts of appropriate isotype controls (Santa Cruz) were used as negative controls. The primary Abs were visualized by fluorescein isothiocyanate (FITC)-conjugated or phycoerythrin (PE)-conjugated F(ab′)2 fragment secondary Abs (Abcam, Cambridge, UK). For intracellular staining, cells were fixed with 4% paraformaldehyde (PFA) by incubation for 15 min at room temperature, permeabilized with 0.1% Triton X-100 for 5 min, washed and stained as described above. Quantitative evaluation of apoptosis was performed by flow cytometry after double staining method using FITC-conjugated Annexin V (AV)/Propidium Iodide (PI) apoptosis detection kit (Marine Biological Laboratory Woods Hole, MA). Reported data are referred to AV-positive apoptotic cells. Acquisition was performed on a FACSCalibur (BD Immunocytometry Systems, San Jose, CA) and data were analyzed using the Cell Quest Pro software (BD Immunocytometry Systems).

### Immunofluorescence experiments

An indirect immunofluorescence assay was developed on SH-SY5Y cells and primary hippocampal neurons, as previously described [44]. Cells were fixed with 4% PFA in PBS for 30 min at 4°C or permeabilized with acetone/methanol 1/1 (v/v) for 10 min at 4°C and then were incubated for 30 min at 25°C in the blocking buffer (2% BSA in PBS, containing 5% glycerol and 0.2% Tween 20). After washing three times with PBS, cells were incubated for 1 h at 4°C with rabbit anti-human ERα MC-20 polyclonal Ab (Santa Cruz), mouse anti-human ERβ 1531 monoclonal Ab (Santa Cruz), and mouse anti-MAP2 monoclonal Ab (Sigma). FITC-conjugated anti-mouse IgG (γ-chain specific, Sigma) were then added and incubated at 4°C for 30 min. After further washings, cells were counterstained with trypan blue dye to distinguish dead cells (excluded from our analysis) from living cells. For actin microfilament detection, samples were stained with fluorescein-phalloidin (Sigma) at 37°C for 30 min. For nuclear staining, the dye Hoechst 33258 (Sigma) was used. Samples were analyzed with an Olympus U RFL microscope (Olympus, Hamburg, Germany).

### Western blot analysis

Cells lysates were prepared as previously described [45]. Western blot was performed with antibodies to: LC3-II (rabbit anti-human LC3-II polyclonal Ab, Cell Signaling Technology, Beverly, MA); p-ERK1/2 (mouse anti-human monoclonal Ab, Cell Signaling); p-p38 (mouse anti-human monoclonal Ab, Cell Signaling). After incubation with peroxidase-conjugated goat anti-rabbit or anti-mouse IgG (Bio-Rad Laboratories, Richmond, CA, USA), the reactions were developed by SuperSignal West Pico chemiluminescent Substrate (Pierce, Rockford, IL). The membranes were re-probed with anti mouse anti-β-actin monoclonal Ab (Amersham, Gent, Belgium). Proteins were quantified by densitometry analysis of the autoradiograms (GS-700 Imaging Densitometer, Bio-Rad).

### Knock down ERα by siRNA

SH-SY5Y were seeded (2×10^4^ cells/dish) in 60 mm dish in DMEM, containing serum and antibiotics. Twenty-four hours after seeding, cells were transfected with GeneSolution siRNA (QIAGEN Sciences, Germantown, MD), according to the manufacturer's instructions, using 5 nM Smart pool siRNA ERα. As experimental control, cells were also transfected with 5 nM of non-silencing siRNA (AllStars Negative Control, QIAGEN). After transfection, cells were treated with H_2_O_2_ for five days as reported above (CMP treatment) and the effect of transfection was verified by flow cytometry analysis by using anti-human ERα MC-20 polyclonal Ab.

### Statistical analyses

The two-tailed Mann-Whitney U test and χ^2^ test were performed. Statistical significance was set at *P*<0.01. Flow cytometry data were statistically evaluated using the Kolmogorov-Smirnov test [46] according to the Cell Quest Pro software guide (BD Immunocytometry Systems) and a D/s ratio ≥15 was accepted as significant in the experimental condition used. Data were expressed as mean ± SD.

## Supporting Information

Figure S1 **mERα and filamentous actin in rat neurons.** mERα (green) and filamentous actin (red) are shown. Note the partial fragmentation of actin filaments in CMP treated cells. No yellow staining is detectable in merge pictures, suggesting the absence of colocalization. Ctr: untreated cells, magnification 5000×. CMP treated cells are shown at low magnification (1200×) in order to display mER positivity in long dendritic protrusions.
(TIF)

Figure S2 **Cell cycle analysis.** Cell cycle distribution was evaluated by flow cytometric analysis in untreated and CMP stress treated SH-SY5Y cells. (**A**) Pie charts show the distribution of cells in each phase of the cell cycle: S phase (white), G2/M (grey) and G0/G1 (dark grey), obtained from three independent experiments. (**B**) Representative quadrant plot graphs are shown. Numbers indicate the percentage of viable cells in each phase of the cell cycle. Ctr, untreated cells.
(TIF)
