# Supplementary figures and images for: Cell Surface Estrogen Receptor Alpha Is Upregulated during Subchronic Metabolic Stress and Inhibits Neuronal Cell Degeneration
Source: PLoS One. 2012 Jul 31;7(7):e42339. doi: 10.1371/journal.pone.0042339 (PMC3409197; doi:10.1371/journal.pone.0042339)

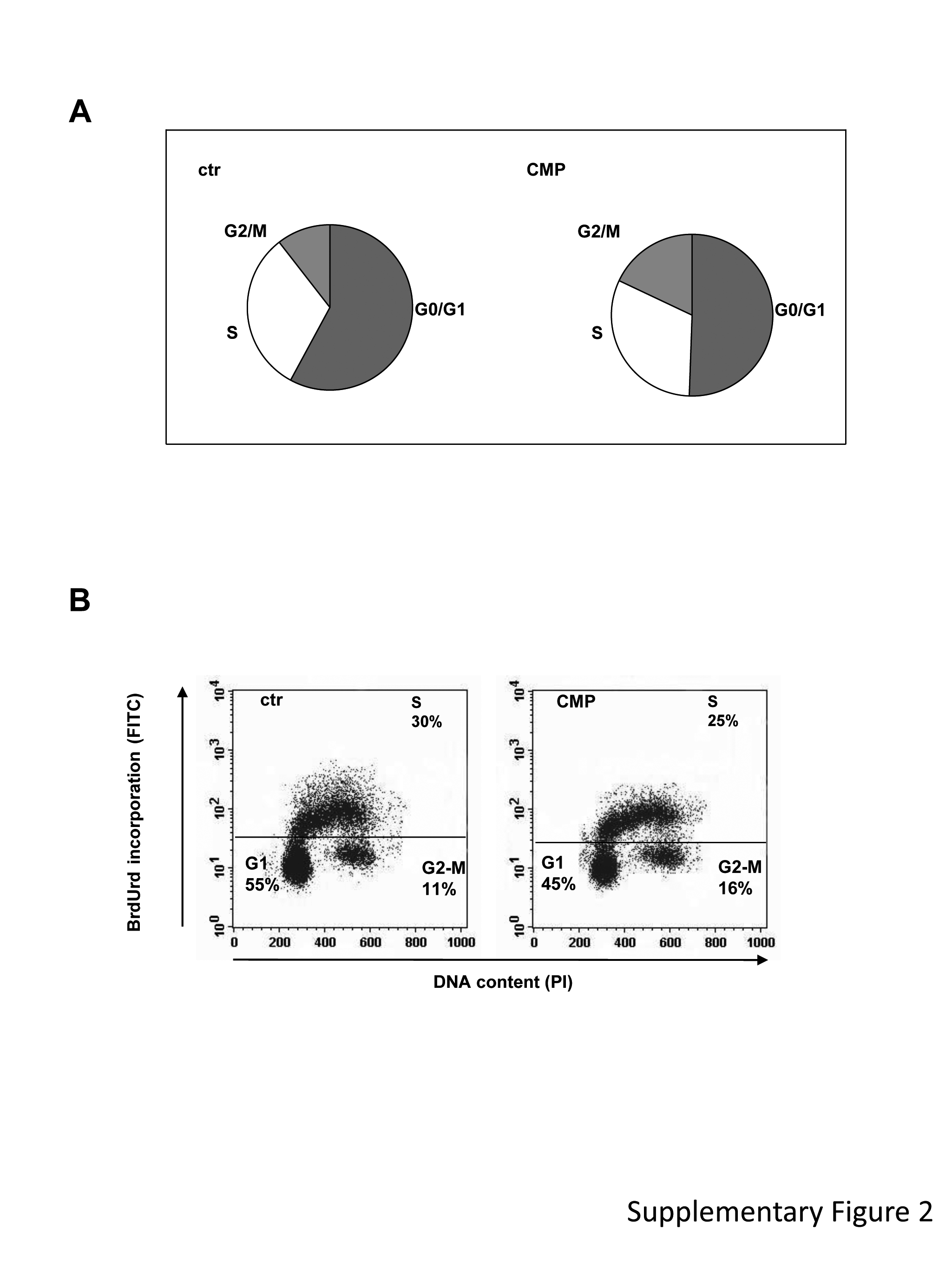

Supplement: Figure S2 — Cell cycle analysis. Cell cycle distribution was evaluated by flow cytometric analysis in untreated and CMP stress treated SH-SY5Y cells. (A) Pie charts show the distribution of cells in each phase of the cell cycle: S phase (white), G2/M (grey) and G0/G1 (dark grey), obtained from three independent experiments. (B) Representative quadrant plot graphs are shown. Numbers indicate the percentage of viable cells in each phase of the cell cycle. Ctr, untreated cells. (TIF) [file pone.0042339.s002.tif]
